# The Complete Plastomes of Five Hemiparasitic Plants (*Osyris wightiana*, *Pyrularia edulis*, *Santalum album*, *Viscum liquidambaricolum*, and *V. ovalifolium*): Comparative and Evolutionary Analyses Within Santalales

**DOI:** 10.3389/fgene.2020.00597

**Published:** 2020-06-16

**Authors:** Xiaorong Guo, Changkun Liu, Guangfei Zhang, Wenhua Su, Jacob B. Landis, Xu Zhang, Hengchang Wang, Yunheng Ji

**Affiliations:** ^1^Institute of Ecology and Geobotany, Yunnan University, Kunming, China; ^2^CAS Key Laboratory for Plant Diversity and Biogeography of East Asia, Kunming Institute of Botany, Chinese Academy of Sciences, Kunming, China; ^3^Department of Botany and Plant Sciences, University of California, Riverside, Riverside, CA, United States; ^4^CAS Key Laboratory of Plant Germplasm Enhancement and Specialty Agriculture, Wuhan Botanical Garden, Chinese Academy of Sciences, Wuhan, China; ^5^Yunnan Key Laboratory for Integrative Conservation of Plant Species with Extremely Small Population, Kunming Institute of Botany, Chinese Academy of Sciences, Kunming, China

**Keywords:** parasitism, plastome degradation, gene loss, pseudogenization, Cervantesiaceae, Santalaceae, Viscaceae

## Abstract

Most species of Santalales (the sandalwood order) are hemiparasites, including both facultative and obligate hemiparasites. Despite its rich diversity, only a small fraction of the species in the sandalwood order have sequenced plastomes. The evolution of parasitism–associated plastome reduction in Santalales remains under-studied. Here, we report the complete plastomes of three facultative hemiparasites (*Pyrularia edulis*, Cervantesiaceae; *Osyris wightiana*, and *Santalum album*, Santalaceae), and two obligate hemiparasites (*Viscum liquidambaricolum* and *Viscum ovalifolium*, Viscaceae). Coupled with publicly available data, we investigated the dynamics of plastome degradation in Santalales hemiparasites. Our results indicate that these hemiparasites can be characterized by various degrees of plastome downsizing, structural rearrangement, and gene loss. The loss or pseudogenization of *ndh* genes was commonly observed in Santalales hemiparasites, which may be correlated to the lifestyle shift from photoautotroph to hemiparasitism. However, the obligate hemiparasites did not exhibit a consistently higher level of gene loss or pseudogenization compared to facultative hemiparasites, which suggests that the degree of plastome reduction is not correlated with the trophic level facultative or obligate hemiparasitism. Instead, closely related taxa tend to possess highly similar plastome size, structure, and gene content. This implies the parasitism-associated plastome degradation in Santalales may evolve in a lineage-specific manner.

## Introduction

Chloroplast, which possess an independent genome (the plastome), is the key organelle in plant cells for photosynthesis and carbon assimilation, as well as the biosynthetic pathways of starch, fatty acids, pigments, and amino acids (Palmer, 1985; Neuhaus and Emes, 2000; Daniell et al., 2016). Due to the essential roles of chloroplast in green plants, their plastomes are characterized by high conservatism in terms of genome size, structure, gene content, and organization (Daniell et al., 2016). A typical angiosperm plastome encodes 113 unique genes arranged in a quadripartite structure, consisting of a large single copy (LSC, 80–90 kbp) region and a small single copy (SSC, 16–27 kbp) region separated by a pair of inverted repeats (IRs, 20–30 kbp) regions (Wicke et al., 2011). Nevertheless, the lifestyle transition from autotrophy to parasitism leads to varying degrees of plastome modification, including decreasing size, physical or functional loss of genes, and structural rearrangement (reviewed by Neuhaus and Emes, 2000; Wicke and Naumann, 2018). As a result, the plastomes of parasitic plants are significantly divergent from their autotrophic relatives (Krause, 2008; Wicke et al., 2013, 2016; Petersen et al., 2015; Frailey et al., 2018; Schneider et al., 2018; Shin and Lee, 2018; Wicke and Naumann, 2018).

There are approximately 4,500 parasitic species documented in 20 families of flowering plants (Nickrent, 1997, 2002; Heide–Jørgensen, 2008), and parasitism likely has evolved at least 11 or 12 times in angiosperms (Barkman et al., 2007; Westwood et al., 2010). Hemiparasitic plants, different from holoparasitic plants (non-photosynthetic parasites) that obtain all nutrition and energy from the host plants, comprise more than 90% of plant parasites and are capable of photosynthesis (Nickrent, 1997; Heide–Jørgensen, 2008; Tešitel, 2016). They hence display a lower degree of plastome degradation than holoparasitic plants, and their plastomes are often within the size range of regular angiosperm plastomes (Wicke and Naumann, 2018).

Hemiparasitic plants can be further divided into facultative and obligate hemiparasites: an obligate cannot survive without exploiting a host, while a facultative does not rely on its host throughout its entire life cycle (Nickrent, 1997; Heide–Jørgensen, 2008). A previous study revealed that facultative hemiparasites may have relatively slight plastome degradation compared with obligate hemiparasites (Petersen et al., 2015). However, the currently available sequenced plastomes of hemiparasitic plants only represent a small fraction of the species diversity. Thus, it remains uncharacterized to what extent their plastomes are divergent, and whether facultative/obligate parasitism is correlated with different levels of plastome degradation.

Comparative and phylogenetic analysis of parasitic plastomes can provide insight into the evolutionary reduction concomitant with the lifestyle shift from autotrophy to parasitism (Wicke et al., 2016). Under a phylogenetic framework, reconstructing an evolutionary pathway of plastome degradation in relation to parasitism becomes feasible (Wicke and Naumann, 2018). The sandalwood order (Santalales) consists of 18 families, approximately 160 genera and 2,200 species (Nickrent et al., 2010; Su et al., 2015), which cover all lifestyles from autotrophy to hemiparasitism and holoparasitism (Nickrent, 1997, 2002; Der and Nickrent, 2008; Su et al., 2015). Most species in Santalales are hemiparasites, including both facultative and obligate (Nickrent, 1997, 2002; Heide–Jørgensen, 2008). Therefore, it provides an ideal system to investigate the reductive plastome evolution in hemiparasitic plants.

In this study, we sequenced and assembled the complete plastomes of three facultative hemiparasites: *Pyrularia edulis* (Cervantesiaceae), *Osyris wightiana*, and *Santalum album* (Santalaceae), and two obligate hemiparasites: *Viscum liquidambaricolum*, and *Viscum ovalifolium* (Viscaceae) in Santalales using a genome skimming approach (Straub et al., 2012). We aim to (1) characterize the newly sequenced plastomes, (2) determine structural shifts, gene loss and/or pseudogenization events in hemiparasites by comparing with autotrophic lineages, and (3) investigate the extent and progression of the plastome reduction in both facultative and obligate hemiparasites.

## Materials and Methods

### Plant Sampling, DNA Extraction, and Illumina Sequencing

Specimen and leaf tissues of *P. edulis*, *O. wightiana*, *S. album*, *V. liquidambaricolum*, and *V. ovalifolium* were sampled with vouchers deposited in the herbarium of Kunming Institute of Botany, CAS (information see Supplementary Table S1). Genomic DNA was extracted from *ca.* 50 mg of silica gel dried leaves using a modified CTAB method (Doyle and Doyle, 1987). Purified DNA was sheared to fragments with an average length of 350 bp, followed by ligation of adaptors for library amplification according to the manufacturer’s guideline (Illumina, San Diego, CA, United States). The shotgun library for each species was sequenced using a 2 × 150 Illumina HiSeq 2500 system at BGI (Wuhan, Hubei, China).

### Plastome Assembly, Annotation and Comparison

Original shotgun reads were filtered using the FASTX–Toolkit^1^ to remove adaptors and reads with ambiguous bases. Both reference-based and *de novo* strategies were employed to assemble the plastomes. Firstly, the clean reads were assembled using CLC genome assembler v. 4.0β (CLC Inc., Aarhus, Denmark) with default parameter setting. The plastome of *Osyris alba* was used as reference to *P. edulis*, *O. wightiana* and *S. album*, and the plastome of *Viscum album* was selected as reference to both *V. ovalifolium* and *V. liquidambaricolum.* Genbank accessions of the reference plastomes are provided in Supplementary Table S2. All contigs were aligned to the reference by BLAST searches. The plastid contigs were organized according to the reference and connected with overlapping terminal sequences to yield the entire plastome. Secondly, the program NOVOPlasty v2.7.0 (Dierckxsens et al., 2017) was used for *de novo* assembly of plastomes with the k–mer size set at 31. The plastid *rbcL* gene of *Taxillus chinensis* (KY996492) was used as the seed to recover the plastomes. The plastomes of a given species recovered from different assembly strategies were compared using Geneious V10.2 (Kearse et al., 2012) to validate the accuracy of assembly.

Annotation of plastid genes were performed with Dual Organellar Genome Annotator database (Wyman et al., 2004). All protein-coding genes were checked with a BLAST search against the NCBI protein database. The protein-coding genes with one or more frame shift mutations or premature stop codons were annotated as pseudogenes. Start and stop codons and intron/exon boundaries for protein-coding genes were checked manually. Genes putatively annotated as transfer RNA (tRNA) were further verified by tRNAscan-SE 1.21 (Schattner et al., 2005) with default parameters. All fully annotated plastomes were deposited in the NCBI GenBank database under the accession number MK675807–MK675811 (Supplementary Table S1).

The published plastomes of Santalales (Petersen et al., 2015; Su and Hu, 2016; Li et al., 2017; Yang et al., 2017; Liu et al., 2018; Shin and Lee, 2018; Zhu et al., 2018; Jiang et al., 2019; Chen et al., 2020) were downloaded from NCBI GenBank (Supplementary Table S2). Together with the newly generated plastomes, the gene content, boundaries of IRs/LSC and IRs/SSC of Santalales hemiparasites were compared using Geneious V10.2 (Kearse et al., 2012). Any putative gene losses detected in the newly generated plastomes were further verified by extracting intact sequences of the corresponding genes from the autotrophic *Erythropalum scandens* plastome and conducting local BLAST searches against the sequencing reads of each species. To investigate structural rearrangement in Santalales plastomes, the plastomes were progressively aligned by the multiple genome alignment software Mauve 2.3.1 (Darling et al., 2010), with *E. scandens* as the reference.

### Phylogenetic Analysis

A phylogenetic tree was inferred with complete plastome DNA sequences. The plastomes of *Colobanthus quitensis* (KT737381) and *Nyssa sinensis* (KX904873) were selected as the outgroup because of the close relationships of Santalales to Caryophyalles and Cornales (Der and Nickrent, 2008; Su et al., 2015). Plastome sequences were aligned using MAFFT (Kazutaka and Standley, 2013), and manually edited where necessary. The most appropriated model of sequence substitution for plastomes (GTR + G) was selected using Modeltest v3.7 (Posada, 1998) with the Akaike information criterion (Posada and Buckley, 2004). We treated the whole plastome as a single inherited unit. Because RAxML can accommodate only one DNA substitution model, we used PartitionFinder v. 2.1.1 (Lanfear et al., 2017) to examine whether the selected mode is commonly suitable for LSC, SSC, and IRs, with the “greedy” search algorithm. We used maximum-likelihood analysis (ML) to reconstruct phylogenetic relationships. The ML analysis was performed using RAxML–HPC BlackBox v8.1.24 (Stamatakis, 2006; Miller et al., 2010) with 10 independent ML searches conducted and the branch support determined by computing 1,000 non-parametric bootstrap (BS) replicates.

## Results

### Features of the Newly Sequenced Plastomes

Illumina paired-end sequencing generated over 30 million clean reads for each species. The mean coverage of the plastome sequencing was 6,518.67X for *O. wightiana*, 573.28X for *P. edulis*, 4314.96X for *S. album*, 134.61X for *V. liquidambaricolum*, and 592.48X for *V. ovalifolium*. The high levels of sequencing coverage guaranteed the accuracy of plastome assembly (Supplementary Table S3). For each species, the reference–based and *de novo* strategies generated almost identical plastomes, with only slight differences in plastome sizes (<5 bp).

**FIGURE 1:**
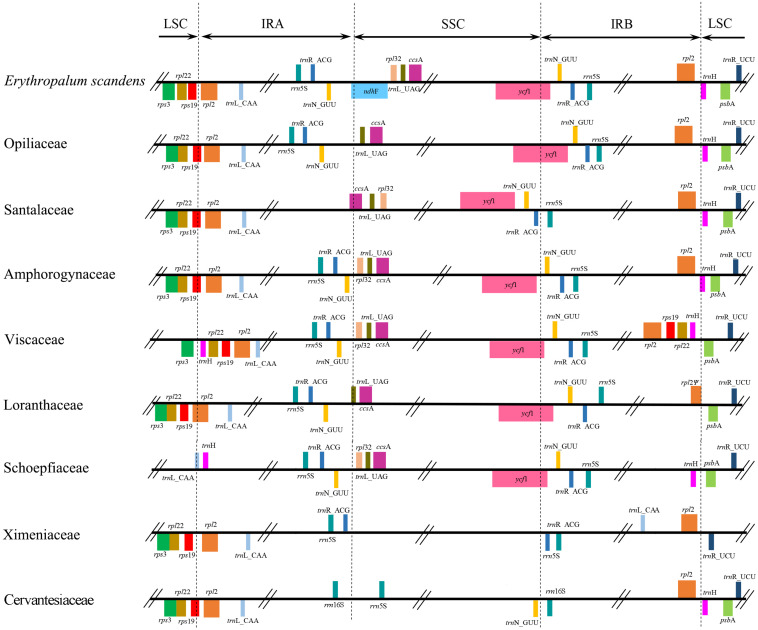
Inverted repeats expansion and contraction dynamics among Santalales plastomes.

Similar to other Santalales hemiparasites, the five newly sequenced plastomes had a typical quadripartite structure, consisting of a pair of IRs, a LSC, and a SSC (Figure 1). The size of these plastomes ranged from 128,601 bp (*V. liquidambaricolum*) to 147,544 bp (*O. wightiana*), with the overall GC content varying from 36.1% (*V. liquidambaricolum* and *V. ovalifolium*) to 38.3% (*P. edulis*) (Table 1). Among the five plastomes, the total gene numbers as well as protein–coding, rRNA, and tRNA genes that constituted each are all slightly different (Table 2).

**TABLE 1:** Comparison of size and GC content (GC%) of complete plastomes, LSC, IR, SSC, coding and non-coding regions among Santalales hemiparasites and the autotrophic outgroup.

		Plastome		LSC		IR		SSC		Coding regions		Non-coding regions	
							
Species*	Lifeform*^,^**	Size (bp)	GC (%)	Size (bp)	GC (%)	Size (bp)	GC (%)	Size (bp)	GC (%)	Size (bp)	GC (%)	Size (bp)	GC (%)
*Arceuthobium sichuanense*	OH	107,526	34.9	65,171	30.1	21,045	42.3	265	26.4	57,987	40.0	49,539	28.8
*Schoepfia jasminodora*	FH	118,743	38.1	84,168	36.1	12,406	47.9	9,763	30.7	70,052	40.8	48,691	34.3
*Schoepfia fragrans*	FH	120,188	38.1	85,643	36.1	12,381	47.9	9,783	30.6	70,089	40.7	50,099	34.4
*Taxillus chinensis*	OH	121,363	37.3	70,357	34.7	22,462	43.0	6,082	26.2	75,421	39.6	45,942	33.5
*Taxillus nigrans*	OH	121,419	37.4	70,181	34.8	22,569	43.0	6,100	26.2	74,727	39.7	46,692	33.8
*Scurrula parasitica*	OH	121,750	37.2	70,270	34.5	22,687	42.9	6,106	25.9	75,603	39.6	46,147	33.4
*Dendrophthoe pentandra*	OH	122,451	36.3	72,451	33.6	22,118	42.2	5,764	25.0	70,262	40.3	52,189	30.9
*Taxillus sutchuenensis*	OH	122,562	37.3	70,630	34.7	22,915	42.8	6,102	26.2	75,292	39.7	47,270	33.6
*Loranthus tanakae*	OH	123,397	36.9	69,522	34.8	23,076	42.2	7,723	24.4	69,905	40.6	53,492	32.1
*Tolypanthus maclurei*	OH	123,581	36.8	72,952	34.3	22,185	42.4	6,259	26.0	75,765	39.6	47,816	32.3
*Scurrula notothixoides*	OH	123,810	37.3	71,448	34.7	23,101	42.9	6,160	26.4	52,652	41.8	71,158	34.1
*Helixanthera parasitica*	OH	124,881	36.5	73,043	33.8	22,752	42.3	6,334	25.5	75,359	39.4	49,522	32.1
*Malania oleifera*	FH	125,050	38.2	76,387	35.0	24,324	43.2	15	40.0	68,012	41.5	57,038	34.2
*Viscum crassulae*	OH	126,064	36.4	73,226	33.6	22,105	43.4	8,628	24.0	78,219	39.3	47,845	31.5
*Macrosolen tricolor*	OH	126,617	37.6	71,893	35.2	24,702	42.4	5,320	25.8	76,219	40.0	50,398	34.0
*Macrosolen sp.*	OH	128,459	37.3	73,347	34.8	22,960	43.3	9,192	27.3	70,900	40.9	57,559	32.9
*Viscum liquidambaricolum*	OH	128,601	36.1	73,831	33.1	23,041	43.2	8,688	23.7	77,414	39.5	51,187	30.8
*Viscum coloratum*	OH	128,746	36.3	73,686	33.4	23,215	43.1	8,630	24.3	76,295	39.8	52,451	31.1
*Viscum album*	OH	128,921	36.4	73,893	33.5	23,198	43.2	8,632	24.8	76,120	39.9	52,801	31.3
*Viscum ovalifolium*	OH	129,465	36.1	74,348	33.2	23,203	43.1	8,711	24.2	76,210	39.9	53,255	30.8
*Pyrularia sinensis*	FH	130,015	37.3	83,917	35.0	19,316	40.7	7,466	46.3	67,533	41.0	62,482	33.3
*Viscum yunnanense*	OH	130,721	35.8	75,844	32.8	22,894	43.3	9,089	22.6	70,452	40.8	60,269	30.2
*Viscum minimum*	OH	131,016	36.2	75,814	33.3	23,094	43.2	9,014	24.2	78,115	39.6	52,901	31.1
*Pyrularia edulis*	FH	132,808	38.3	74,811	36.1	24,548	42.8	8,901	31.2	76,747	40.9	56,061	34.6
*Dendrotrophe varians*	OH	140,666	37.8	81,684	35.5	24,056	43.7	10,870	29.7	76,484	40.7	64,182	34.4
*Santalum album*	FH	144,101	38.0	83,802	35.9	24,511	43.1	11,277	31.4	78,274	40.8	65,827	34.7
*Osyris alba*	FH	147,253	37.7	84,601	35.6	24,340	43.1	13,972	31.2	78,206	40.7	69,047	34.2
*Champereia manillana*	FH	147,461	37.4	83,505	35.3	28,075	41.9	7,806	27.9	78,095	40.5	69,366	33.9
*Osyris wightiana*	FH	147,544	37.6	84,569	35.5	24,447	43.0	14,081	31.1	78,173	40.6	69,371	34.2
*Erythropalum scandens*	Autotroph	156,154	38.0	84,799	36.2	26,394	42.8	18,567	32.3	90,488	40.3	65,666	34.8
*Ximenia americana*	FH	156,834	36.8	87,816	34.4	32,691	40.4	3,636	27.5	84,385	40.0	72,449	33.0

*The species are arranged from the smallest to the largest overall plastome by size. **Life form: FH, facultative hemiparasite; OH, obligate hemiparasite.

**TABLE 2:** Comparison of the plastome gene content of Santalales hemiparasites with *Erythropalum scandens.*

Species	Lifeform*	Total plastid genes	Potentially functional genes	Functional protein–encoding genes	tRNA	rRNA	Deleted genes	Pseudogenes
*Erythropalum scandens*	Autotroph	113	113	79	30	4	0	0
*Osyris alba*	FH	110	101	67	30	4	3	9
*Osyris wightiana*	FH	110	101	67	30	4	3	9
*Santalum album*	FH	108	101	67	30	4	5	7
*Champereia manillana*	FH	105	100	66	30	4	8	5
*Dendrotrophe varians*	OH	105	101	67	30	4	8	4
*Ximenia americana*	FH	103	102	68	30	4	10	1
*Pyrularia edulis*	FH	102	100	67	29	4	11	2
*Schoepfia fragrans*	FH	101	101	68	29	4	12	0
*Schoepfia jasminodora*	FH	101	101	68	29	4	12	0
*Viscum yunnanense*	OH	101	97	64	29	4	12	4
*Viscum minimum*	OH	100	98	66	28	4	13	2
*Viscum album*	OH	100	97	65	28	4	13	3
*Viscum coloratum*	OH	100	96	64	28	4	13	4
*Viscum liquidambaricolum*	OH	100	97	65	28	4	13	3
*Viscum ovalifolium*	OH	100	97	65	28	4	13	3
*Viscum crassulae*	OH	99	98	66	28	4	14	1
*Pyrularia sinensis*	FH	97	94	62	28	4	16	3
*Taxillus sutchuenensis*	OH	96	94	63	27	4	17	2
*Scurrula notothixoides*	OH	96	91	60	27	4	17	5
*Macrosolen sp.*	OH	96	94	63	27	4	17	2
*Macrosolen tricolor*	OH	96	95	64	27	4	17	1
*Taxillus chinensis*	OH	95	94	63	27	4	18	1
*Scurrula parasitica*	OH	95	94	63	27	4	18	1
*Tolypanthus maclurei*	OH	94	93	64	25	4	19	1
*Dendrophthoe pentandra*	OH	94	92	63	25	4	19	2
*Loranthus tanakae*	OH	94	90	61	25	4	19	4
*Taxillus nigrans*	OH	94	92	62	26	4	19	2
*Helixanthera parasitica*	OH	93	92	63	25	4	20	1
*Malania oleifera*	FH	85	84	58	22	4	28	1
*Arceuthobium sichuanense*	OH	84	81	54	23	4	29	3

*Life form: FH, facultative hemiparasite; OH, obligate hemiparasite.

The plastomes of *O. wightiana* and *S. album* retained 101 potentially functional genes (Table 2). The pseudogenization of *infA*, *ndhA*–*E*, *ndhG*–*H*, and *ndhK*, as well as the deletion of *ndhF*, *ndhI*, and *ndhJ*, were observed in *O. wightiana*. In *S. album*, *infA*, *ndhB*–*E*, *ndhG*, and *ndhK* were identified as pseudogenes, and *ndhA* and *ndhH* had been deleted. Although the loss of all *ndh* genes except for *ndhB* (pseudogenized) was detected in *P. edulis*, *V. liquidambaricolum*, and *V. ovalifolium*, the additional loss of *infA*, *trnG-UCC*, and *trnV-UAC*, as well as the pseudogenization of *ccsA*, were only identified in *V. liquidambaricolum* and *V. ovalifolium* (Supplementary Table S4). Also, the loss of *trnR-ACG* and pseudogenization of *matK* was observed in *P. edulis* and *V. ovalifolium*. These gene losses were confirmed using corresponding sequences of *E. scandens* for local BLAST searches against the Illumina reads of each species. As the result of gene loss and pseudogenization, a total of 100, 97 and 95 putatively functional genes were observed in *P. edulis*, *V. liquidambaricolum*, and *V. ovalifolium*, respectively.

### Comparison of Plastome Size and GC Content of Santalales Hemiparasites

Based on the plastomes sequenced in this study, as well as those reported previously, we compared the plastome size and GC content of Santalales hemiparasites with those of *E. scandens* (Table 1). All hemiparasite plastomes, except for that of *Ximenia americana* (156,834 bp), are smaller than the 156,154 bp of the autotrophic outgroup. The LSC length of the Santalales hemiparasites ranged from 65,171 bp (*Arceuthobium sichuanense*) to 87,816 bp (*X. americana*), whereas the length of IR and SSC regions varied from 12,381 bp (*Schoepfia fragrans*) to 32,691 bp (*X. americana*), and 15 bp (*Malania oleifera*) to 14,081 bp (*O. wightiana*), respectively. These regions of the hemiparasites (except for *X. americana*), were reduced in size to varying degrees compared to those of *E. scandens* (84,799 bp in LSC, 26,394 bp in each IR, and 18,576 bp in SSC). Also, the hemiparasite plastomes had varying GC content from 34.9% (*A. sichuanense*) to 38.3% (*P. edulis*). In each plastome, LSC, SSC, and IR regions had an uneven distribution of GC content. Specifically, the IR regions had the highest GC (40.4–47.9%), followed by LSC (30.1–36.1%). The SSC region exhibited the lowest GC content (23.7–40.0%).

### Structural Shifts in Santalales Hemiparasitic Plastomes

**FIGURE 2:**
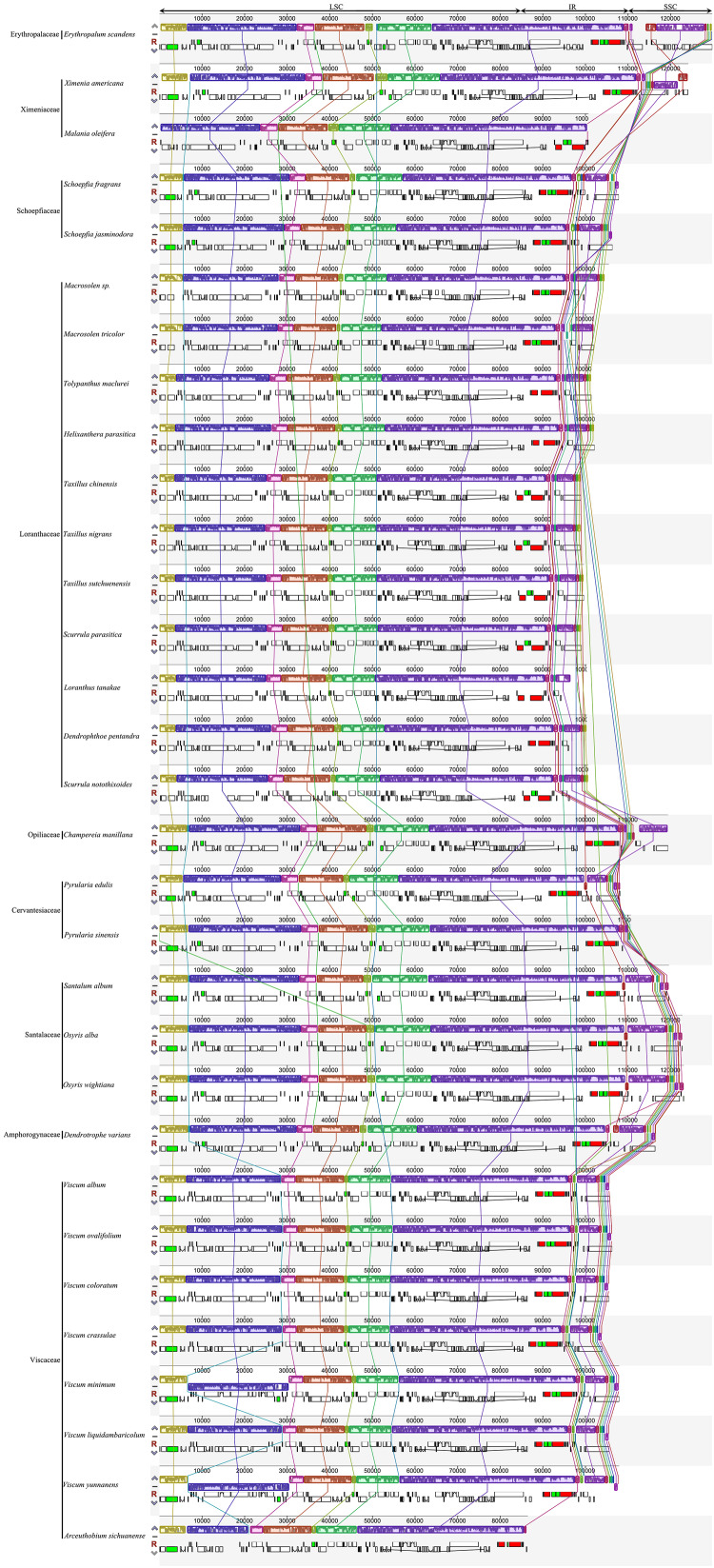
Multiple Mauve alignment of Santalales plastomes.

The junctions of IR/LSC and IR/SSC in the plastomes of Santalales hemiparasites are variable (Figure 1). The SSC region has been extremely contracted to 15 bp in *M. oleifera* (Ximeniaceae), and 265 bp in *A. sichuanense* (Viscaceae). A significant IR contraction to the *ycf2* at the IR/LSC junctions was observed in the plastomes of Schoepfiaceae. The IR/LSC boundaries of all Loranthaceae species are in *rpl2*, and the IR/SSC junctions fall into *trnL-UAG* and *ycf1*. Within Santalaceae, the IR falls into *rps19* in LSC, and in *ccsA* and the *trnR*-*trnN* intergenic spacer in SSC. The plastomes of Cervantesiaceae and *C. manillana* (Opiliaceae) share the same IR/LSC boundaries with species of Santalaceae, while their IRb/SSC junction shifted to the intergenic spacer between *trnN-GUU* and *rrn5S*, and *trnL-UAG* and *trnN-GUU*. The expansion of IR regions in Viscaceae reached *rpl22-trnH-rps3* cluster in the IR/LSC boundaries, and their IR/SSC fall into *ycf1*, as well as the intergenic spacer between *trnN-GUU* and *rpl32*. The result of the multiple plastome alignment is illustrated in Figure 2. Compared with the autotrophic *E. scandens* plastome, no structural rearrangement was found in the LSC and IR regions of Santalales hemiparasites. However, multiple rearrangements were found in the IR/SSC boundaries, and SSC regions in species of Amphorogynaceae, Cervantesiaceae, Santalaceae, and Viscaceae.

### Comparison of Plastome Gene Content and Organization

**FIGURE 3:**
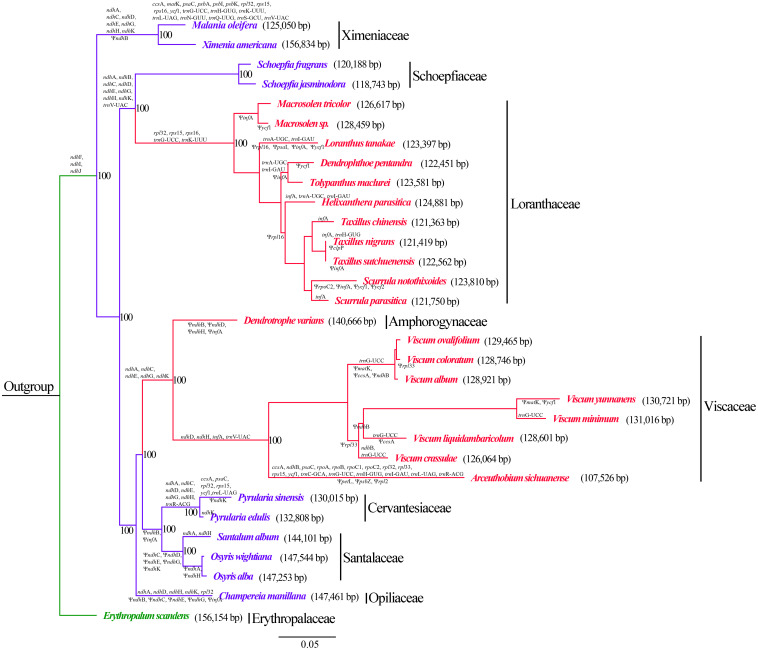
Phylogenetic relationships within Santalales hemiparasites. The branches are labeled with specific gene loss (genes above branches) and pseudogenization (genes under branches). Plastome size of each species are shown in the parentheses behind the botanical name. The number at each node is the maximum-likelihood bootstrap percentage. The branch lengths are proportional to the substitution rate. Green lineages: autotrophy outgroup; blue lineages: facultative hemiparasites; red lineages: obligate hemiparasites.

The examined Santalales hemiparasites had divergent gene content in their plastomes (Table 2 and Supplementary Table S4 and Figure 3). The plastomes of *Dendrotrophe varians*, *O. alba*, *O. wightiana*, *S. album*, *S. fragrans*, and *Schoepfia jasminodora* encoded the highest number of potentially functional genes with a total of 101, including 67 protein-coding, 30 tRNA, and 4 rRNA genes. Comparatively, the lowest number of putatively functional genes was observed in *A. sichuanense*, which has 54 protein-coding, 23 tRNA, and 4 rRNA genes. The functional or physical losses of the plastid-encoded NAD(P)H–dehydrogenase (NDH) complex is commonly shared by all Santalales hemiparasites, while the loss of plastid *infA* was identified in Cervantesiaceae, Loranthaceae, Opiliaceae, Santalaceae, and Viscaceae. In addition, the deletion of *trnV–UAC* genes was shared by species of Loranthaceae, Schoepfiaceae, Viscaceae, and Ximenaceae. The further loss of *trnG-UCC* was observed in Loranthaceae, Viscaceae, and Ximeniaceae. The family specific losses of *rpl32*, *rps15*, *rps16*, and *trnK-UUU* were observed in Loranthaceae and Ximeniaceae, and the further deletion of essential photosynthetic genes (*psaC*, *psbA*, *I*, *K*, *L* and *M*, *petB*, and *ccsA*) was identified in Ximeniaceae.

### Phylogenetic Analysis

Maximum-likelihood analysis (Figure 3) resolved the autotrophic taxa *E. scandens* (Erythropalaceae) as the early diverging clade of the sandalwood order, and recovered the hemiparasites in three major clades (BS = 100%). Notably, the obligate hemiparasites did not form a monophyletic group, with obligate hemiparasitism evolving at least twice from facultative parasitism. Of those hemiparasites, Ximeniaceae (*M. oleifera* and *X. Americana*) was resolved as the first diverging branch. Within the second clade, Loranthaceae were recovered as monophyletic (BS = 100%), which was sister to Schoepfiaceae (BS = 100%). Within the third clade, *C. manillana* (Opiliaceae) is sister to clade comprised of Santalaceae + Cervantesiaceae, *D. varians* (Amphorogynaceae), and Viscaceae. The predicted gene deletions and gene inactivation shared by specific branches is shown in the tree topology. We also compared the plastome size among close species. The results indicate that closely related taxa possess similar-sized plastomes as well as relatively analogous gene content.

## Discussion

### Plastome Downsizing and Structural Shifts

The lifestyle transition from autotrophy to parasitism often triggers reductive plastome evolution (Wicke and Naumann, 2018). Compared with the autotrophic *E. scandens*, the plastomes of Santalales hemiparasites have reduced in size by approximately 6–31%. Among them, *X. americana* possessed the largest plastome (156,834 bp). Contrarily, in the most reduced plastome (107,526 bp) of *A. sichuanense*, a total of 29 genes have been lost. The observation of more genes missing and a smaller sized plastome (Table 2), suggests that gene loss can be partly responsible for the plastome downsizing in Santalales hemiparasites.

We also found that the decrease of plastome size in Santalales is accompanied with a length reduction of non-coding regions, including intergenic regions between functional genes and introns (Table 1). A similar phenomenon has been shown in the hemiparasitic *Cuscuta* species (McNeal et al., 2007). A previous study proposed that the downsizing of non-coding regions have evolved to prompt the efficiency of energy and nutrition utilization, particularly in disadvantageous environments (Wu et al., 2009). Because of their nutritional reliance on host plants, reduction of non-coding regions may reinforce their fitness to the partial heterotrophic lifestyle.

Inverted repeat expansion and contraction frequently occur in angiosperm plastomes. These events often cause the lineage-specific gain or loss of a small number of genes in the IR/LSC, and IR/SSC junctions (Plunkett and Downie, 2000; Chumley et al., 2006). Although large-scale IR expansions and contractions are only observed in a few autotrophic species (Chumley et al., 2006), dramatic IR shifts commonly occur in the plastomes of both holoparasites (Bungard, 2004; Maier et al., 2007; McNeal et al., 2007; Krause, 2008; Naumann et al., 2016; Samigullin et al., 2016; Sidonie et al., 2016; Schneider et al., 2018) and hemiparasites (Li et al., 2013, 2017; Su and Hu, 2016; Cho et al., 2018; Frailey et al., 2018; Liu et al., 2018; Park et al., 2018; Shin and Lee, 2018; Jiang et al., 2019; Chen et al., 2020). Similarly, significant IR and SSC contractions were found in Schoepfiaceae, *M. oleifera* and *A. sichuanense* (Figure 1), respectively. Moreover, multiple rearrangements occurred in the SSC regions in hemiparasitic plastomes of Amphorogynaceae, Cervantesiaceae, Santalaceae, and Viscaceae (Figure 2). This suggests that the evolution of parasitism has caused prominent structural changes in the plastomes of some Santalales hemiparasites.

### Missing and Pseudogenized Genes

The lifestyle transition from autotrophy to parasitism always leads to prevalent gene losses from the plastomes (Neuhaus and Emes, 2000; Wicke et al., 2013, 2016; Wicke and Naumann, 2018). Compared to autotrophic species, Santalales hemiparasites have experienced significant gene deletion and pseudogenization (Table 2). The series of reductive mutations involves *ndh* genes, *infA*, *matK*, *rpl32*, *rpl33*, *rps15*, *rps16*, *ycf1*, as well as several photosynthesis-associated genes (*psaC*, *psbA*, *I*, *K*, *L* and *M*, *petB*, and *ccsA*) and tRNAs (Figure 3 and Supplementary Table S4).

The *ndh* genes encode subunits of the NADH dehydrogenase complex, which mediates photosystem I electron recycle and facilitates chlororespiration in plant cells (Yamori and Shikanai, 2016). In general, the *ndh* genes are the earliest functional losses in the plastomes of hemiparasites (Maier et al., 2007; Wicke et al., 2013, 2016; Wicke and Naumann, 2018). In Santalales hemiparasites, all eleven *ndh* (*A*–*K*) genes have either been deleted or pseudogenized, suggesting that the plastid NDH pathway is not essential in these species. It is noteworthy that the physical or functional losses of these plastid–encoded *ndh* genes does not occur exclusively in parasitic or heterotrophic plants. The degeneration of *ndh* loci have been observed in a wide spectrum of autotrophic lineages, such as Gnetales and Pinaceae of gymnosperm (Wakasugi et al., 1994; McCoy et al., 2008; Braukmann et al., 2009; Wu et al., 2009), the early diverging eudicots (Sun et al., 2016, 2017), the eudicot families Cactaceae (Sanderson et al., 2015) and Geraniaceae (Blazier et al., 2011), the aquatic monocot *Najas* (Peredo et al., 2013), and epiphytic orchids (Chang et al., 2006; Lin et al., 2015, 2017). Given the independent loss of this pathway in many plant groups, Frailey et al. (2018) proposed that the plastid *ndh* genes may have been selected against in these photoautotrophic lineages. Also, a previous study revealed the NDH complex–related genes have been concomitantly deleted from plastid and nuclear genomes in some orchids, and proposed that the mutation in photoautotrophic plants may increase the possibility to evolve a heterotrophic life history (Lin et al., 2017). Given that all *ndh* genes are retained in their autotrophic relative (*E. scandens*), we hypothesize that the degradation of these genes from plastomes of Santalales hemiparasites may be associated with the lifestyle shift from photoautotroph to hemiparasitism. The eco-physiological consequences of the functional and physical losses of the plastid NDH pathway in Santalales hemiparasites, especially under stress conditions, merit to be furtherly examined (Chen et al., 2020).

Another commonly degraded plastid gene in Santalales is *infA*; this mutation was detected in all Santalales hemiparasites except for Ximeniaceae and Schoepfiaceae. The loss or pseudogenization of *infA* occurs in the plastomes of many holoparasitic plants (Wicke et al., 2011, 2013, 2016; Wicke and Naumann, 2018). With respect to hemiparasitic plants, reduction of this gene has been exclusively observed in Santalales (Petersen et al., 2015; Li et al., 2017; Yang et al., 2017; Liu et al., 2018; Shin and Lee, 2018; Zhu et al., 2018; Jiang et al., 2019; Chen et al., 2020). Given that pseudogenization or deletion of *infA* gene has also been identified in a wide diversity of photoautotrophic plant lineages (Shinozaki et al., 1986; Millen et al., 2001; Ahmed et al., 2012), previous studies revealed that *infA* is likely to be one of the most mobile plastid genes in flowering plants, which are often transferred to and maintained in the nucleus (Millen et al., 2001; Ahmed et al., 2012). Therefore, it is possible that *infA* has been likewise transferred to the nucleus, and the degradation of this gene in Santalales hemiparasites may not be correlated to the evolution of parasitism.

Besides the functional or physical losses of *ndh* loci and *infA*, further plastome degradations were found in some Santalales hemiparasites. For example, the loss of *rps15*, *rps16*, and *rpl32* was commonly shared by all Loranthaceae and Ximeniaceae species (Figure 3). Similarly, losses of these plastid ribosomal protein–coding genes have been observed in diverse lineage of autotrophic angiosperms (e.g., Saski et al., 2005; Jansen et al., 2007; Wicke et al., 2011; Sabir et al., 2014; Park et al., 2015; Schwarz et al., 2015). A line of evidence indicates that such genes are transferred to the nucleus in these plants (Park et al., 2015). One possibility is that the plastid ribosomal protein-coding genes have likewise been transferred to the nucleus, and the reduction of these genes in Santalales hemiparasites may not be resulted from the evolution of parasitism. In addition, losses of tRNA genes were commonly observed in Santalales hemiparasites. With smaller size, the import of tRNAs from the cytoplasm may be relatively easy (Wicke and Naumann, 2018). Therefore, plastid tRNA genes may be dispensable even at an early parasitic stage. Following the degradation of *ndh* genes, those non-essential genes such as tRNAs tend to be deleted from hemiparasitic plastomes.

Plastid genes that encode photosystem (*psa*/*psb*), cytochrome complex (*pet*), and heme attachment (*ccsA*) are key genes involved in photosynthesis (Wicke et al., 2011; Wicke and Naumann, 2018). Loss or pseudogenization of those core photosynthesis genes had been commonly found in holoparasitic angiosperms (e.g., Funk et al., 2007; McNeal et al., 2007; Wicke et al., 2016; Wicke and Naumann, 2018). According to the model of reductive plastome evolution associated with parasitism (Wicke et al., 2016; Wicke and Naumann, 2018), functional losses of the photosynthesis–associated genes (e.g., *psa/psb* genes, *pet* genes, *ccsA*, *cemA*) may occur around the boundary to holoparasitism. However, the observation of *ccsA* pseudogenization in Viscaceae species, as well as the loss of *psaC*, *psbA*, *I*, *K*, *L* and *M*, *petB*, and *ccsA* in *M. oleifera*, imply that the degradation of these core photosynthesis genes might initiate in hemiparasites that still heavily rely on photosynthesis. Because of their great importance for photosynthesis, further studies are needed to clarify whether these genes have been functionally transferred to the nuclear genome (Chen et al., 2020).

### Lineage–Specific Plastome Degradation

A previous study suggested that the varying nutritional dependences on a host may influence the reductive evolution of hemiparasitic plastomes (Petersen et al., 2015). Distinct from facultative hemiparasite, an obligate hemiparasite cannot complete the life cycle without coming into contact with a host (Heide–Jørgensen, 2008). As a result, their plastomes may be expected to possess a higher level of degradation. Similar to a previous study (Chen et al., 2020), we found that the range of plastome size of the Santalales facultative hemiparasites (118,743–156,834 bp) largely overlaps the range for obligated ones (107,526–140,666 bp). We also observed that some facultative and obligate plants exhibited the same degree of gene deletion and pseudogenization. For instance, *Pyrularia* species (facultative) and *D. varians* (obligate hemiparasites) maintained almost identical gene content. In addition, a total of 28 genes has been deleted from the facultative hemiparasite, *M. oleifera*. The level of plastome degradation is higher than the majority of obligate hemiparasites (except for *A. sichuanense*) in Santalales. This suggests that obligate hemiparasitism may not lead to a higher level of physical or functional loss of plastid genes than facultative one. Therefore, the degree of plastome degradation may not be correlated with the lifeform of facultative or obligate.

The relationships within the sandalwood order recovered in this study received high support values (Figure 3), which are highly congruent with previous studies (Der and Nickrent, 2008; Nickrent et al., 2010; Su et al., 2015; Chen et al., 2020). Based on the phylogenetic relationships recovered in this study, we found that closely related taxa, for instance, within the same genera or family tend to possess high level of similarity in plastome size, structure, and gene content. We hence hypothesize that plastome degradation in Santalales hemiparasites may evolve in a lineage–specific manner.

## Data Availability Statement

The newly sequenced plastomes in this study were deposited in the NCBI GenBank database under the accession numbers MK675807–MK675811 (Supplementary Table S1).

## Author Contributions

YJ, XG, HW, and WS designed the research. XG, CL, and GZ collected and analyzed the data. XG and YJ wrote the manuscript. HC, XZ, and JL discussed the results and revised the manuscript.

## Conflict of Interest

The authors declare that the research was conducted in the absence of any commercial or financial relationships that could be construed as a potential conflict of interest.

## Footnotes

1 http://hannonlab.cshl.edu/fastx_toolkit

## Supplementary Material

TABLE S1 Voucher information and GenBank accession.

TABLE S2 Lifeform and GenBank accession of published Santalales plastomes.

TABLE S3 Summary of Illumina sequencing of the five Santalales hemiparasites.

TABLE S4 Comparison of plastome gene content among Santalales hemiparasites.
