# Supplementary material for: The Complete Plastomes of Five Hemiparasitic Plants (Osyris wightiana, Pyrularia edulis, Santalum album, Viscum liquidambaricolum, and V. ovalifolium): Comparative and Evolutionary Analyses Within Santalales
Source: Front Genet. 2020 Jun 16;11:597. doi: 10.3389/fgene.2020.00597 (PMC7308561; doi:10.3389/fgene.2020.00597)
Supplement: TABLE S1 — Voucher information and GenBank accession. [file Table_1.DOCX]

**Table S1.** Voucher information and GenBank accession.

| Species | Lifeform | Locality | Voucher | Accession number |
| --- | --- | --- | --- | --- |
| *Osyris wightiana* | Facultative hemiparasitism | Gengma County, Yunnan, China | Y. Ji 2017114 | MK675807 |
| *Pyrularia edulis* | Facultative hemiparasitism | Gengma County, Yunnan, China | Y. Ji 2017111 | MK675808 |
| *Santalum album* | Facultative hemiparasitism | South China Botanical Garden, Guangzhou, China | Y. Ji 2018002 | MK675809 |
| *Viscum liquidambaricolum* | Obligate hemiparasitism | Tengchong County, Yunnan, China | L. Yang 010 | MK675810 |
| *V. ovalifolium* | Obligate hemiparasitism | Tengchong County, Yunnan, China | L. Yang 008 | MK675811 |
