# Supplementary material for: The Complete Plastomes of Five Hemiparasitic Plants (Osyris wightiana, Pyrularia edulis, Santalum album, Viscum liquidambaricolum, and V. ovalifolium): Comparative and Evolutionary Analyses Within Santalales
Source: Front Genet. 2020 Jun 16;11:597. doi: 10.3389/fgene.2020.00597 (PMC7308561; doi:10.3389/fgene.2020.00597)
Supplement: TABLE S2 — Lifeform and GenBank accession of published Santalales plastomes. [file Table_2.DOCX]

**Table S2.** Lifeform and GenBank accession of published Santalales plastomes.

| Species | Lifeform | Family | Genbank accession |
| --- | --- | --- | --- |
| *Dendrotrophe varians* | Obligate hemiparasitism | Amphorogynaceae | NC_039391 |
| *Dendrophthoe pentandra* | Obligate hemiparasitism | Loranthaceae | MN414174 |
| *Helixanthera parasitica* | Obligate hemiparasitism | Loranthaceae | MG808038 |
| *Loranthus tanakae* | Obligate hemiparasitism | Loranthaceae | MN414178 |
| *Macrosolen sp.* | Obligate hemiparasitism | Loranthaceae | MN414161 |
| *Macrosolen tricolor* | Obligate hemiparasitism | Loranthaceae | MN414162 |
| *Scurrula notothixoides* | Obligate hemiparasitism | Loranthaceae | MH220878 |
| *Scurrula parasitica* | Obligate hemiparasitism | Loranthaceae | MH101514 |
| *Taxillus chinensis* | Obligate hemiparasitism | Loranthaceae | KY996492 |
| *Taxillus sutchuenensis* | Obligate hemiparasitism | Loranthaceae | NC_036307 |
| *Taxillus nigrans* | Obligate hemiparasitism | Loranthaceae | MH095982 |
| *Tolypanthus maclurei* | Obligate hemiparasitism | Loranthaceae | NC_042257 |
| *Arceuthobium sichuanense* | Obligate hemiparasitism | Viscaceae | MN414160 |
| *Viscum album* | Obligate hemiparasitism | Viscaceae | NC_028012 |
| *Viscum coloratum* | Obligate hemiparasitism | Viscaceae | NC_035414 |
| *Viscum crassulae* | Obligate hemiparasitism | Viscaceae | NC_027959 |
| *Viscum minimum* | Obligate hemiparasitism | Viscaceae | NC_027829 |
| *Viscum yunnanens* | Obligate hemiparasitism | Viscaceae | MN414173 |
| *Champereia manillana* | Facultative hemiparasitism | Opiliaceae | KY436366 |
| *Malania oleifera* | Facultative hemiparasitism | Ximeniaceae | MG799332 |
| *Ximenia americana* | Facultative hemiparasitism | Ximeniaceae | MN414175 |
| *Osyris alba* | Facultative hemiparasitism | Santalaceae | NC_027960 |
| *Schoepfia jasminodora* | Facultative hemiparasitism | Schoepfiaceae | KX775962 |
| *Schoepfia fragrans* | Facultative hemiparasitism | Schoepfiaceae | MN414171 |
| *Pyrularia sinensis* | Facultative hemiparasitism | Cervantesiaceae | MN414172 |
| *Erythropalum scandens* (outgroup) | Autotroph | Erythropalaceae | NC_036759 |
