# Supplementary material for: The Complete Plastomes of Five Hemiparasitic Plants (Osyris wightiana, Pyrularia edulis, Santalum album, Viscum liquidambaricolum, and V. ovalifolium): Comparative and Evolutionary Analyses Within Santalales
Source: Front Genet. 2020 Jun 16;11:597. doi: 10.3389/fgene.2020.00597 (PMC7308561; doi:10.3389/fgene.2020.00597)
Supplement: TABLE S3 — Summary of Illumina sequencing of the five Santalales hemiparasites. [file Table_3.DOCX]

**Table S3.** Summary of Illumina sequencing of the five Santalales hemiparasites.

| Species | No. of clean reads | Plastid-like reads (percentage) | Sequencing coverage (×) |
| --- | --- | --- | --- |
| *Osyris wightiana* | 30,710,530 | 6,399,293(20.84%) | 6,518.672 |
| *Pyrularia edulis* | 31,374,872 | 562,776 (1.79%) | 573.275 |
| *Santalum album* | 31,036,076 | 4,235,943 (13.65%) | 4,314.964 |
| *Viscum liquidambaricolum* | 30,014,270 | 115,696 (0.39%) | 134.613 |
| *V. ovalifolium* | 31,404,168 | 509,221(1.62%) | 592.480 |
